# Gender dimorphism in IgA subclasses in T2-high asthma

**DOI:** 10.1007/s10238-022-00828-x

**Published:** 2022-04-25

**Authors:** Gilda Varricchi, Remo Poto, Bianca Covelli, Gaetano Di Spigna, Maria Rosaria Galdiero, Gianni Marone, Loredana Postiglione, Giuseppe Spadaro

**Affiliations:** 1 Department of Translational Medical Sciences, University of Naples Federico II, 80131 Naples, Italy; 2 Center for Basic and Clinical Immunology Research (CISI), University of Naples Federico II, 80131 Naples, Italy; 3 World Allergy Organization (WAO) Center of Excellence, 80131 Naples, Italy; 4 Institute of Experimental Endocrinology and Oncology (IEOS), National Research Council, 80131 Naples, Italy

**Keywords:** Asthma, Gender dimorphism, IgA, IgA1, IgA2, Immunoglobulins

## Abstract

Immunoglobulin A (Chan in J Allergy Clin Immunol 134:1394–14014e4, 2014), the second most abundant immunoglobulin in serum, plays an important role in mucosal homeostasis. In human serum, there are two subclasses of IgA, IgA1 (≅ 90%) and IgA2 (≅ 10%), transcribed from two distinct heavy chain constant regions. This study evaluated the serum concentrations of total IgA, IgA1, and IgA2, and total IgG, IgG1, IgG2, IgG3, and IgG4 in T2-high asthmatics compared to healthy controls and the presence of gender-related variations of immunoglobulins. Total IgA levels were increased in asthmatics compared to controls. Even more marked was the increase in total IgA in male asthmatics compared to healthy male donors. IgA1 were increased only in male, but not in female asthmatics, compared to controls. Concentrations of IgG2, but not IgG1, IgG3, and IgG4, were reduced in asthmatics compared to controls. IgG4 levels were reduced in female compared to male asthmatics. In female asthmatics, IgA and IgA1 levels were increased in postmenopause compared to premenopause. IgA concentrations were augmented in mild, but not severe asthmatics. A positive correlation was found between IgA levels and the age of patients and an inverse correlation between serum concentrations of IgA2 and IgE in asthmatics. A positive correlation between total IgA or IgA2 and IgG2 was found in asthmatics. These results highlight a gender dimorphism in IgA subclasses in male and female T2-high asthmatics. More adequate consideration of immunological gender disparity in asthma may open new opportunities in personalized medicine by optimizing diagnosis and targeted therapy.

**Supplementary Information:**

The online version contains supplementary material available at 10.1007/s10238-022-00828-x.

## Introduction

Accumulating evidence indicates that gender has an impact on gene expression in various inflammatory diseases [2–6]. Males and females differ in their immunological responses to antigens and show distinctions in innate and adaptive immune responses [7]. Certain immunological sex differences are present throughout life, whereas others are only apparent before puberty and after menopause, suggesting that both genes and hormones are involved. It is important to acknowledge gender differences when considering the prevalence of various diseases between males in females. Under the Severe Asthma Research Program (SARP), the gender difference in asthma incidence, prevalence, and severity was assessed [8]. Between the age of 4 and 14 years, asthma was more prevalent in boys compared to girls. However, after puberty, asthma became more prevalent and severe in women [9]. Interestingly, after menopause, asthma becomes more severe in males [10]. Moreover, female patients experience more symptoms when compared to males, resulting in a poorer quality of life [9]. Even though gender-related differences in asthma prevalence and clinical features have been described, the mechanisms underlying this phenomenon have been incompletely elucidated.

Besides gender, aging profoundly influences the immune system [11–14]. In humans, the serum concentrations of IgG and IgA generally tend to increase slightly with age [15]. In postmenopausal women, changes in the immune system have been attributed to estrogen deprivation [16]. Sex hormones modulate both humoral and cell-mediated immune responses [17].

Immunoglobulin A [1] is the most produced antibody class in the body (≅ 60 mg/kg/day) and the predominant antibody in mucosal surfaces, where it plays an important role in mucosal homeostasis [18–20]. At mucosal surfaces, a joining (J) chain is added to IgA when it is synthesized forming primarily dimers [21]. The dimers bind to the poly-Ig receptor (pIgR) expressed at the basolateral side of mucosal epithelial cells before being transported to the apical side of the cells where it is released into the lumen by proteolytic cleavage [22]. Unlike IgA produced at mucosal surfaces, serum IgA is predominantly monomeric and not joined to the J chain or the secretory component [23]. In humans, IgA represents the second most prevalent immunoglobulin in the serum [24, 25]. There are two subclasses of human IgA, IgA1 and IgA2, transcribed from two distinct heavy chain constant regions, with the first one dominating in serum and most tissues and the second mainly secreted at the mucosal surfaces [26, 27]. Human IgA1 and IgA2 are encoded by different genes, *α*1 and *α*2, respectively, located on chromosome 14 [24, 28]. The two IgA subclasses have different stability and effector functions due to highly significant differences in the hinge region and glycosylation patterns [29, 30]. In human serum, the predominant IgA form is monomeric with a subclass distribution of about 90% IgA1 and 10% IgA2 [27, 29]. It has become clear that serum IgA1 has specific immunological functions independent from the role of secretory IgA [29]. Although the pathogenic role of IgE in asthma is well-established [31–33], the complex interplay of IgE, IgG, and IgA subclasses in allergic diseases has been recently reviewed [34].

Asthma is a heterogeneous group of inflammatory respiratory disorders characterized by distinct phenotypes (T2-high and T2-low) and variable clinical course [35–38]. The majority of asthmatic patients fall into the T2-high phenotype characterized by increased IgE and FeNO, hypereosinophilia, and overexpression of Th2 cytokines [35, 37]. T2-low asthma is presumably a heterogeneous condition incompletely understood [36, 38, 39].

To the best of our knowledge, the serum concentrations of IgG1 and IgA2 subclasses in asthma have not been reported so far. The aim of the study was twofold: first, to evaluate the serum concentrations of IgA (IgA1, IgA2) and IgG (IgG1, IgG2, IgG3, IgG4) subclasses in adult T2-high asthmatics compared to age-matched healthy controls; second, to investigate the presence or absence of gender-related variations of serum levels of IgA and IgG subclasses between male and female asthmatics and controls.

## Materials and methods

### Patients and methods

This case-control study was carried out at the Center for Basic and Clinical Immunology Research (CISI) of the Department of Translational Medical Sciences, University of Naples Federico II (Naples, Italy), from September 2020 to October 2021. Forty-three Caucasian asthmatic patients (mean age 46.7 ± 15.8 years) were recruited in the outpatient clinic of the Division of Allergy and Clinical Immunology. Fifty-five volunteers were enrolled as healthy matched controls (mean age 43.8 ± 9.5 years). The study was approved by the Ethics Committee of the University of Naples Federico II, School of Medicine (Prot. 198/18), and informed consent was obtained from all participants prior to collection of blood according to recommendations from the Declaration of Helsinki. All participants were enrolled if adherent to stringent exclusion and inclusion criteria and provided written informed consent to participate in the study. Patients were eligible for enrollment in the study if they were aged 18–70 years and had a clinical diagnosis of asthma according to Global Initiative for Asthma [GINA] 2021 criteria [40]. Key exclusion criteria for both healthy controls and asthmatics were acute and chronic infections, bronchiectasis, primary and secondary immunodeficiencies, autoimmune diseases, malignancies, cystic fibrosis, patient-reported smoking history or the onset of respiratory symptoms after the age of 40 years in current or previous smokers with a smoking history of at least 10 pack-years. None of the asthmatic patients has been or was treated with allergen-specific immunotherapy [41] or monoclonal antibodies anti-IgE, anti-IL-5/IL-5R*α*, or anti-IL-4R*α* [42–44]. Forty out of 43 patients were treated with daily low-dose of inhaled glucocorticoids (ICS) therapy [fluticasone propionate (FP), 100–200 μg or equivalent] plus two additional controllers (e.g., a long-acting *β*2-agonist and/or leukotriene receptor antagonist and/or long-acting muscarinic agonist); 3/43 of patients were treated with daily medium-dose of ICS (FP, 250–500 μg or equivalent); 6/43 of patients were on oral glucocorticoids (mean daily prednisone intake 11.6 mg/die). Table 1 shows the demographic and clinical characteristics of the patients and healthy volunteers included in the study. The following parameters were evaluated: the on-treatment forced expiratory volume in 1 s (FEV_1_, in liters), the score on the Asthma Control Test (ACT) [45], serum IgE, total IgA and subclasses (IgA1 and IgA2), and total IgG and subclasses (IgG1, IgG2, IgG3, IgG4). Pulmonary function test (Quark PTF, COSMED, Pavona di Albano, Italy) was performed according to the American Thoracic Society/European Respiratory Society (ATS/ERS) guidelines [36]. FEV_1_, Forced Vital Capacity (FVC), and FEV_1_/FVC were measured, and the best of three forced maneuvers was recorded. Results were expressed both as absolute values and as a percentage of the predicted values referred to European Respiratory Society (ERS) reference values [35]. The Body Mass Index (BMI) was calculated as body weight [46]/height^2^ (m^2^) [46]. Peripheral blood leukocyte counts were measured using an automated hematology analyzer [47].

**Table 1:** Demographic and clinical characteristics of healthy controls and asthma patients

Characteristics	Healthy controls	Asthma patients	*p-*value
Subjects, *n*^°^	55	43	NA
Age (years)	43.8 ± 9.5	46.7 ± 15.8	0.54
Sex, male/female	32/23	11/32	NA
BMI (Kg/m^2^)	25.79 ± 3.24	26.01 ± 4.46	0.82
Smokers, *n*^°^(%)	None	None	NA
Age of asthma diagnosis	NA	28.4 ± 18.8	NA
Annualized Asthma Exacerbation Rate (AAER)	NA	0.53 ± 1	NA
ACT (score)	NA	16.3 ± 5.2	NA
Allergic Rhinitis, *n*^°^ (%)	None	37 (86.1)	NA
CRSsNP, *n*^°^ (%)	None	13 (30.02)	NA
CRSwNP, n^°^ (%	None	7 (16.3)	NA
FEV_1_ (L/s)	3.72 ± 0.88	2.44 ± 0.82	****
FEV_1_ (%)	106.3 ± 11.74	78.85 ± 21.05	****
FEV_1_/FCV ratio (%)	100 ± 9.44	93.26 ± 13.38	NS
FEF_25–75_ (%)	110 ± 18.5	75.47 ± 36.23	****
ICS use (%)	None	83.7	NA
LABA use (%)	None	74.4	NA
LTRA use (%)	None	44.2	NA
LAMA use (%)	None	4.7	NA
OCS use, *n* (%)	None	6 (14.0)	NA
Dose of OCS (mg/d), PRED equivalent	None	11.6 ± 10.3	NA

*CRSsNP* chronic sinusitis without nasal polyps, *CRSwNP* chronic sinusitis withnasal polyps, *FEV*_*1*_ Forced expiratory volume in the 1st second; *FVC* Forced vital capacity; *FEV*_*1*_*/FCV ratio (%)* actual ratio of the two parameters; *FEF25*_*−75*_ forced expiratory flow between 25 and 75% of FVC; *ICS* Inhaled glucocorticoids; *OCS* Oral glucocorticoids; *LABA* Long-acting *ß*-2 agonist; *LTRA* Leukotriene-receptor antagonist; *LAMA* Long-acting muscarinic agonist; *ACT* asthma control test; *BMI* Body mass index. *PRED* prednisone; *NA* not applicable; *p < 0.05; **p < 0.01; ***p < 0.0005; ****p < 0.0001

### Measurement of serum immunoglobulins

Serum samples from venous blood were stored in aliquots at − 80 °C until tested. Total IgE, IgG and subclasses (IgG1, IgG2, IgG3, IgG4), and IgA were measured by nephelometry using Behring BN™ II System (Siemens Healthcare Diagnostics Ltd, Erlangen, Germany). Calibration was performed using N protein standard SL (OQIM), according to Sanguin nephelometric standard M1590 (based on WHO67/97 reference serum) [48]. The precision evaluation (coefficient of variation: CV) for the measurement of IgG and IgG subclasses was done according to the Clinical and Laboratory Standards Institute (CLSI) EP05-A3 guidelines [49]. The intra-assay CVs ranged from 1.8 to 3.6%. The linearity for the measurement of IgG and IgG subclasses (coefficient of determination: R^2^) was assessed according to the CLSI EP06-A guidelines [50]. The *R*^2^ values ranged from 0.97 to 0.99. IgA1 and IgA2 subclasses were assessed by using the Binding Site SPA_PLUS_® turbidimetric analyzer (The Binding Site, Birmingham, UK). The CVs were 1.4% (IgA1) and 1.7% (IgA2). The correlation coefficients were 0.99 (IgA1) and 0.99 (IgA2). IgE were measured by chemiluminescent immunoassay using Immunolite 2000 (Siemens Healthcare Diagnostics Ltd, Erlangen, Germany). The CV was 2.9%, and the coefficient of correlation was 0.99.

### Statistical analysis

Statistical analysis was performed by using GraphPad Prism 8 software (GraphPad Software, La Jolla, CA, USA). Data are expressed as mean ± standard deviation (SD) of the indicated number of experiments. Values from groups were compared by Student’s *t* test or Mann–Whitney U test based on the parametric or nonparametric distribution of the continuous variables. One-way analysis of variance (ANOVA) followed by Tukey’s post hoc was used for multiple comparisons. For the correlation analyses, Pearson’s correlation method was used. *P* values less than 0.05 were considered significant.

## Results

### Demographic and clinical characteristics of asthma patients and healthy controls

The demographic and clinical characteristics of healthy donors and asthma patients are given in Table 1. The median age was 46.7 ± 15.8 years for asthmatics and 43.8 ± 9.5 years for healthy controls. Thirty-two patients (74.4%) were female and 11 (25.6%) were male. None of healthy controls and asthma patients were smokers. All patients had a T2-high phenotype based on skin test positivity and/or specific IgE (65.2%), hypereosinophilia (34.8%), and increased FeNO levels. Asthmatic patients had an annualized asthma exacerbations rate (AAER) of 0.53 and an ACT score of 16.3 ± 5.2. Allergic rhinitis (86.1%) and chronic rhinosinusitis without nasal polyps (30.2%) and with nasal polyps (CRSwNP) (16.3%) were comorbidities of asthmatics. FEV_1_ in asthmatics was lower in healthy controls (2.44 ± 0.82 L/s *vs.* 3.72 ± 0.88 L/s; *p* < 0.0001).

### Serum concentrations of IgE, IgA1, and IgA2 subclasses in asthma patients and healthy controls

**Fig. 1:**
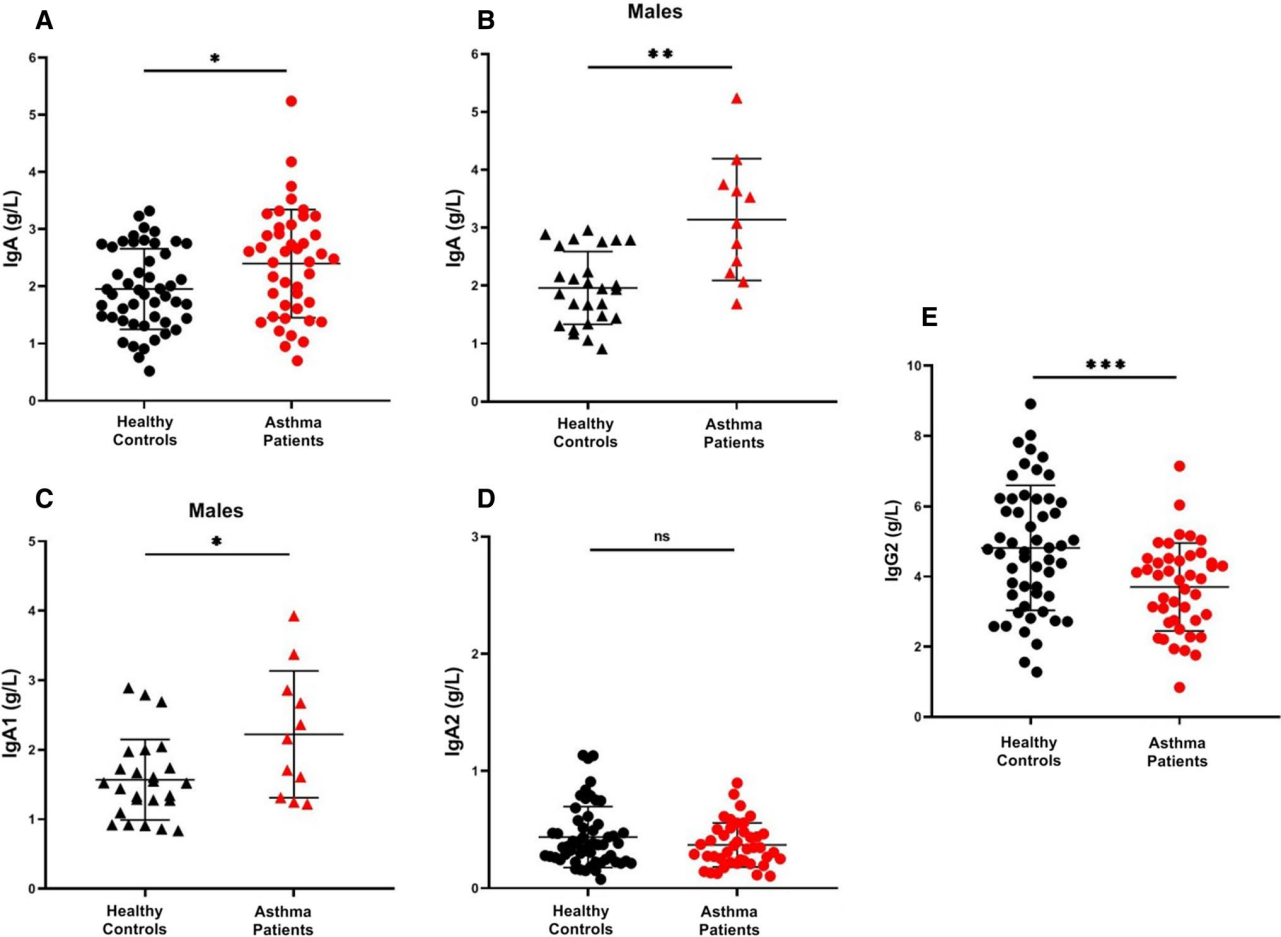
Serum concentrations of IgA in healthy controls and asthma patients (**a**) Serum concentrations of IgA in healthy male controls and asthma patients (**b**) Serum concentration of IgA1 in healthy male controls and asthma patients (**c**) Serum concentrations of IgA2 in healthy controls and asthma patients (**d**) Serum concentrations of IgG2 in healthy controls and asthma patients (**e**) Data are presented as scatter plots with mean ± SD. Significance was tested with two sided Student’s t test. *p < 0.05; **p < 0.01; ***p < 0.001

The serum concentrations of IgE, total IgA, IgA1, and IgA2 subclasses in controls and asthmatics are given in Table 2, which also reports the concentrations of these immunoglobulin classes and subclasses in male and female subjects. As expected, serum IgE concentrations were significantly increased (*p* < 0.0001) in all asthmatic subjects irrespectively of gender (Table 2). Total serum IgA levels were also increased (*p* < 0.05) in asthmatics compared to controls (Fig. 1A). Even more marked (*p* < 0.01) was the increase in total serum IgA in male asthmatics when compared to healthy male donors (Fig. 1B). By contrast, the serum concentrations of IgA were not different between female asthmatics and controls (Table 2).

**Table 2:** Serum concentrations of IgE, IgA, IgA1, and IgA2 subclasses in healthy controls and asthma patients

Laboratory data	Healthy controls	Asthma patients	*p-*value
*Serum total IgE (Log *_*10*_*)*			
Total	1713	2563	****
Male	1613	2565	****
Female	1694	2453	****
Serum IgA (g/L)			
Total	1.95 ± 0.70	2.39 ± 0.94	*
Male	1.96 ± 0.62	3.142 ± 1.05	**
Female	1.94 ± 0.79	2.105 ± 0.83	NS
*Serum IgA1 (g/L)*			
Total	1.530 ± 0.57	1.708 ± 0.74	NS
Male	1.568 ± 0.57	2.223 ± 0.91	*
Female	1.486 ± 0.57	1.526 ± 0.59	NS
*Serum IgA2 (g/L)*			
Total	0.437 ± 0.25	0.369 ± 0.18	NS
Male	0.462 ± 0.29	0.408 ± 0.21	NS
Female	0.398 ± 0.19	0.356 ± 0.17	NS

*NA* not applicable; *NS* not significant. *p < 0.05; **p < 0.01; ****p < 0.0001

In human serum, IgA1 represents the major (≅ 90%) IgA subclass [27]. Therefore, we measured serum IgA1 and IgA2 subclasses in asthmatics and controls. Serum IgA1 did not differ between these two groups. However, when subjects were analyzed by gender, we found that serum IgA1 levels were significantly increased (*p* < 0.05) in male asthmatics compared to controls (Fig. 1C), but not in females (Table 2). IgA2 represents approximately 10% of serum IgA [27]. The serum concentrations of IgA2 did not differ between asthmatics and controls in both males and females (Table 2).

### Serum concentrations of total IgG and subclasses in asthma patients and healthy controls

Serum concentrations of total IgG did not differ between asthmatics and controls in both males and females (Table 3). Similarly, IgG1, IgG3, and IgG4 subclasses did not differ between asthmatics and healthy donors in both males and females (Table 3). By contrast, concentrations of IgG2 were lower in asthmatics when compared to controls (*p* < 0.001) (Fig. 1E). The latter difference was particularly evident in males (*p* < 0.0001) but not in females (Table 3).

**Table 3:** Serum concentrations of IgG and IgG subclasses in healthy controls and asthma patients

Laboratory data	Healthy controls	Asthma patients	*p-*value
*Serum IgG (g/L)*			
Total	10.71 ± 2.1	10.38 ± 2.4	NS
Male	11.50 ± 1.7	10.04 ± 2.1	NS
Female	9.78 ± 2	10.49 ± 2.5	NS
*Serum IgG1 (g/L)*			
Total	7.15 ± 1.4	7.52 ± 2.2	NS
Male	7.44 ± 1.2	7.06 ± 2.7	NS
Female	6.86 ± 1.6	7.67 ± 2.1	NS
*Serum IgG2 (g/L)*			
Total	4.81 ± 1.7	3.70 ± 1.2	***
Male	5.51 ± 1.5	3.92 ± 0.9	****
Female	3.86 ± 1.5	3.62 ± 1.3	NS
*Serum IgG3 (g/L)*			
Total	0.31 ± 0.3	0.34 ± 0.2	NS
Male	0.33 ± 0.3	0.31 ± 0.1	NS
Female	0.27 ± 0.1	0.34 ± 0.2	NS
*Serum IgG4 (g/L)*			
Total	0.89 ± 0.6	0.86 ± 0.9	NS
Male	0.99 ± 0.4	1.5 ± 1.3	NS
Female	0.74 ± 0.7	0.63 ± 0.6	NS

*NS* not significant. *p < 0.05. ****p < 0.0001

### Gender-specific differences of serum immunoglobulins in asthma patients

The previous results highlighted unexpected gender-specific differences in total IgA and IgA1 (Table 2). Therefore, we systematically compared gender-specific differences of all serum immunoglobulins examined (IgE, total IgA, IgA1, IgA2, total IgG, IgG1, IgG2, IgG3, and IgG4) in asthma patients (Table 4).

**Table 4:** Gender-specific differences in asthma patients

Laboratory data	Asthmatic males	Asthmatic females	*p-*value
Serum total IgE (Log _10_)	2565	2453	NS
Serum IgA (g/L)	3.142 ± 1.05	2.105 ± 0.83	***
Serum IgA1 (g/L)	2.223 ± 0.91	1.526 ± 0.59	*
Serum IgA2 (g/L)	0.408 ± 0.21	0.356 ± 0.17	NS
Serum IgG (g/L)	10.04 ± 2.1	10.49 ± 2.5	NS
Serum IgG1(g/L)	7.06 ± 2.7	7.67 ± 2.1	NS
Serum IgG2(g/L)	3.92 ± 0.9	3.62 ± 1.3	NS
Serum IgG3 (g/L)	0.31 ± 0.1	0.34 ± 0.2	NS
Serum IgG4 (g/L)	1.5 ± 1.3	0.63 ± 0.6	*

*NS* not significant. *p < 0.05; ***p < 0.001

**Fig. 2:**
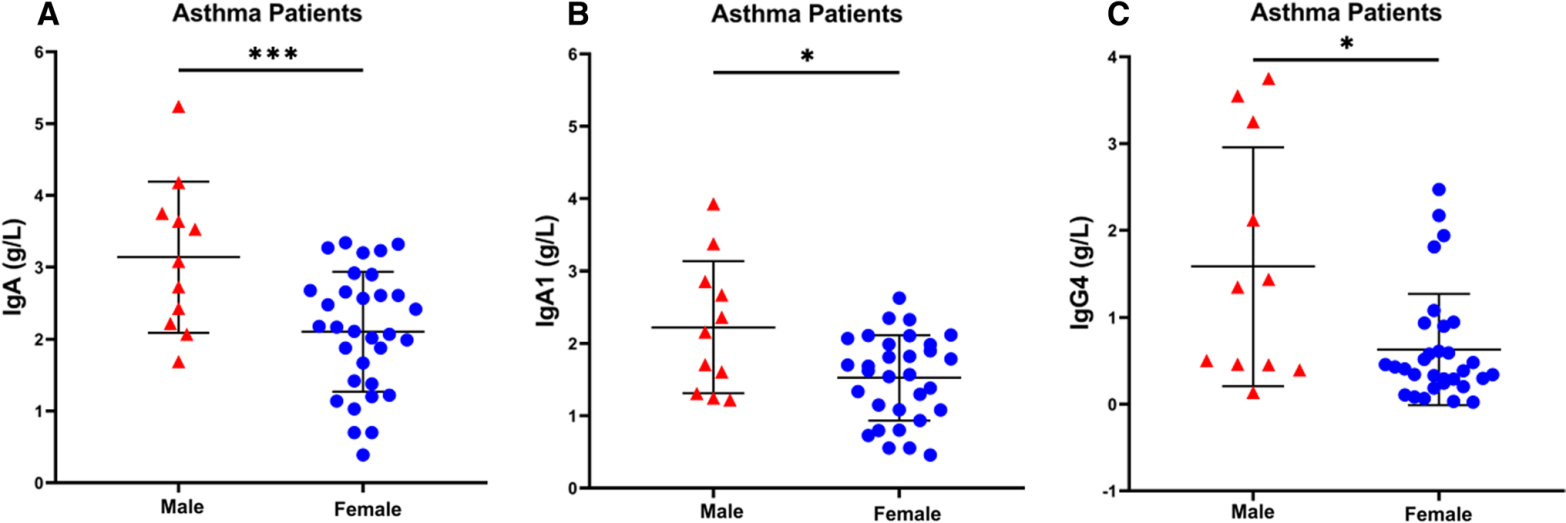
Serum concentration of IgA (**a**), IgA1 (**b**) and IgG4 (**c**) in male and female asthma patients. Data are presented as scatter plots with mean ± SD. Significance was tested with two-sided Student’s t test. *p < 0.05; ***p < 0.001

Figure 2A shows that serum concentrations of total IgA were markedly increased (*p* < 0.001) in males when compared to female asthmatics. Examining serum concentrations of IgA1, we found that asthmatic males had higher values than females (*p* < 0.05) (Fig. 2B). Interestingly, also the concentrations of IgG4 were increased (*p* < 0.05) in male asthmatics compared to females (Fig. 2C). Concentrations of other IgA and IgG subclasses did not differ between male and female asthmatics (Table 4).

### Serum concentrations of IgA pre- and postmenopause in female asthma patients

**Fig. 3:**
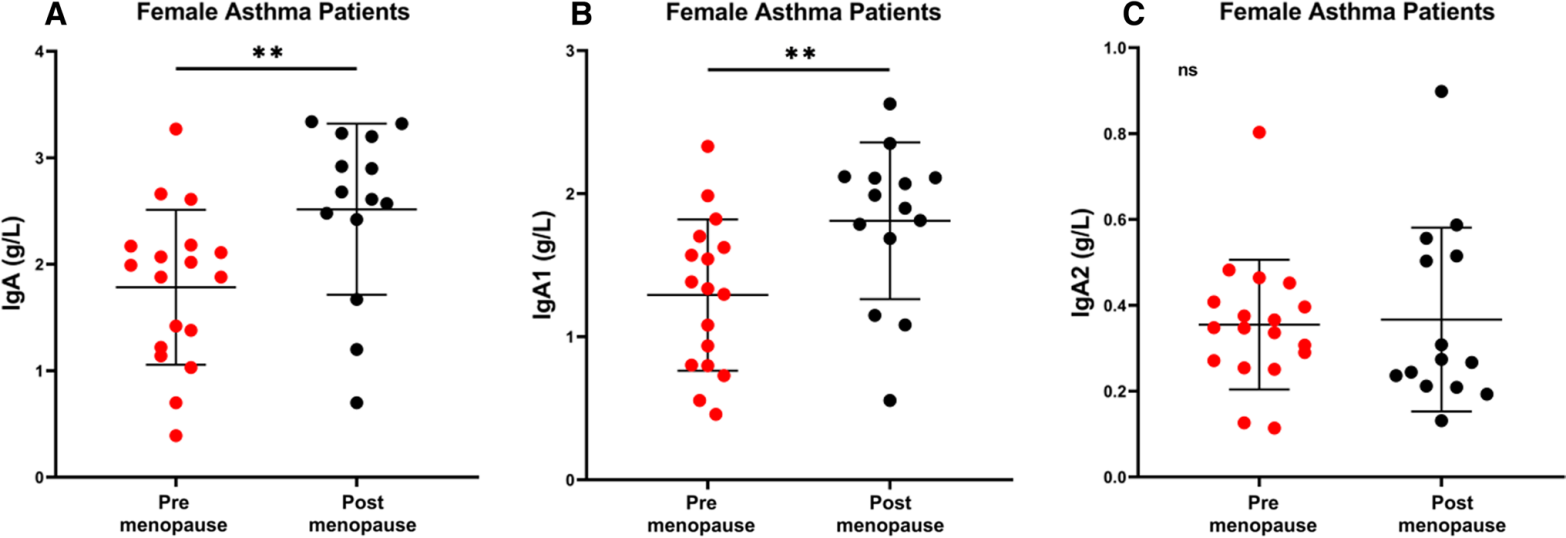
Serum concentrations of IgA (**a**), IgA1 (**b**), and IgA2 (**c**) in pre- (mean age 33.6 ± 10 years) and postmenopause (mean age 60.3 ± 5.1 years) female asthmatic patients. Data are presented as scatter plots with mean ± SD. Significance was tested with two-sided Student’s t test. **p < 0.01

Gender differences in asthma incidence, prevalence, and severity have been started to be appreciated [8]. Moreover, menopause can affect asthma severity [10]. Besides gender, aging profoundly influences the innate and adaptive immune system [11–14]. When we evaluated the effects of menopause on total IgA and IgA subclasses in asthmatics, we found that serum concentrations of total IgA were significantly increased (*p* < 0.01) after menopause (Fig. 3A). Similarly, the serum levels of IgA1 were increased (*p* < 0.01) after menopause (Fig. 3B). The specificity of this observation was supported by the finding that serum concentrations of IgA2 were not affected by menopause in female asthma subjects (Fig. 3C). Similarly, serum levels of IgG and their subclasses and IgE did not differ in controls and asthmatics between pre- and postmenopause (data not shown).

### Correlations between asthma severity and serum concentration of IgA

**Fig. 4:**
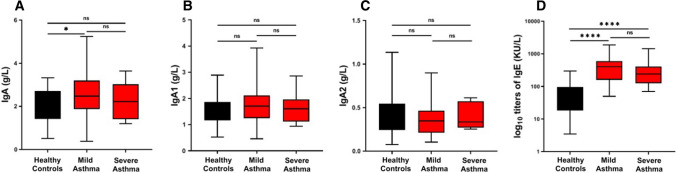
Serum concentrations of IgA (**a**), IgA1 (**b**), IgA2 (**c**) and IgE (**d**) in healthy controls, mild, and severe asthmatics. Data are presented as box plots with medians and interquartile ranges plus whiskers ranging from min to max. Significance was tested using one-way ANOVA followed by Tukey’s post hoc. *p < 0.05; ****p < 0.0001

The alterations of serum concentrations of total IgA and IgA1 in asthmatics prompted us to investigate the possible correlations between asthma severity and the concentrations of these immunoglobulins. Figure 4A shows that serum levels of total IgA were increased in mild, but not in severe asthmatics compared to controls. Serum concentrations of IgA1 in mild and severe asthmatics did not differ from controls (Fig. 4B), whereas IgA2 levels were reduced in both mild and severe asthma patients *vs.* controls (Fig. 4C). As expected, IgE levels were significantly increased (*p* < 0.0001) in both mild and severe asthmatics (Fig. 4D).

### Correlations between serum concentrations of IgA and age of asthmatics and controls

Serum levels of IgG and IgA tend to increase slightly with age [15]. In adult healthy subjects, there was no correlation between serum concentrations of IgA, IgA1, and IgA2 and the age of donors (data not shown). By contrast, we found a positive correlation (*r* = 0.36; *p* < 0.05) between serum levels of total IgA and the age of asthmatics (Supplementary Fig. 1). No significant correlations were found between the age of asthmatics and serum concentrations of both IgA1 and IgA2 (data not shown).

### Correlations between serum concentrations of IgE and IgA subclasses in asthmatics and controls

**Fig. 5:**
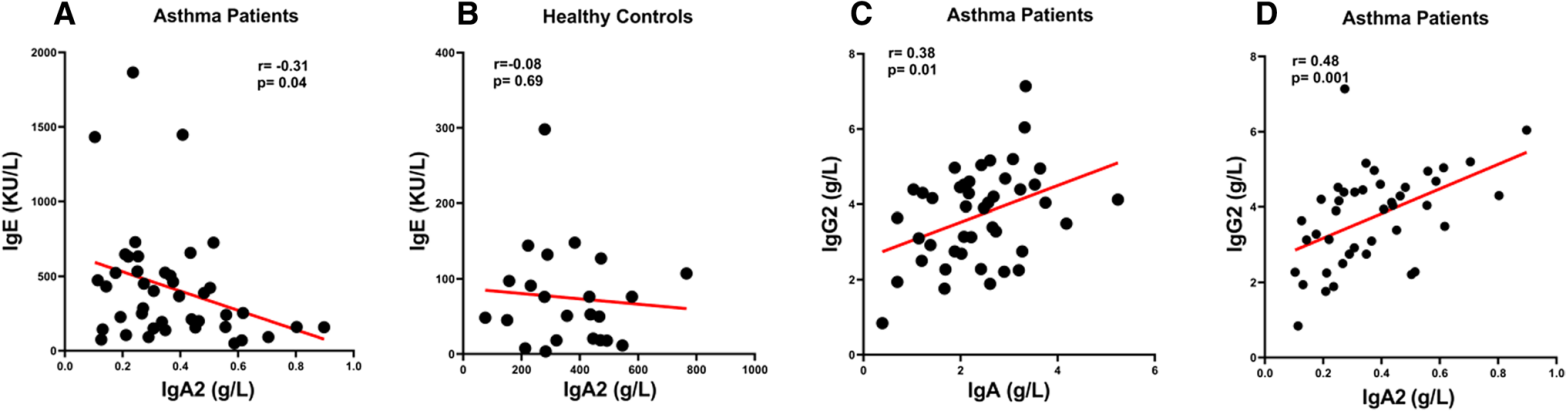
Correlations between serum concentrations of IgA2 and IgE in asthma patients (**a**) and healthy controls (**b**). Correlations between serum concentrations of IgA (**c**) or IgA2 (**d**) and IgG2 in asthma patients. Significance was tested with Pearson’s correlation method. *p < 0.05; ***p < 0.001

The pathogenic role of IgE in asthma and allergic disorders is well-established [31–33]. The role of IgA subclasses in asthma has been recently emphasized [34]. We found an inverse correlation (r = −0.31; *p* < 0.05) between serum concentrations of IgA2 and IgE in asthmatics (Fig. 5A) but not in controls (Fig. 5B). No correlations were found between serum IgE and total IgA and IgA1 in both normal donors and asthmatics (data not shown).

### Correlations between serum concentrations of IgA and IgG subclasses in asthmatics and controls

We investigated possible correlations between serum concentrations of IgA and IgG subclasses in asthmatics and controls. There was no correlation between total IgA and IgG in both controls and asthmatics (data not shown). Similarly, there was no correlation between total IgA and IgG1, IgG2, IgG3, and IgG4 in healthy subjects (data not shown). In asthmatics, there was a correlation between serum concentrations of total IgA and IgG2 (*r* = 0.38; *p* < 0.01) (Fig. 5C). Similarly, there was a correlation (*r* = 0.48; *p* < 0.001) between serum concentrations of IgA2 and IgG2 (Fig. 5D). As expected, we found close correlations between serum levels of total IgG and IgG1 (*r* = 0.84; *p* < 0.0001), IgG2 (*r* = 0.40; *p* < 0.001), IgG3 (*r* = 0.32; *p* < 0.05), but not IgG4 (*r* = 0.09; NS) in asthmatics (Supplementary Fig. 2A, B, C, D). By contrast, we found a positive correlation between total IgG and only IgG1 in the control group (*r* = 0.68; *p* < 0.0001) (Supplementary Fig. 3A, B, C, D).

## Discussion

In this study, we found that serum concentrations of IgE and total IgA were significantly increased in adult patients with T2-high asthma compared to healthy controls. IgA levels were increased in males but not in female asthmatics. Similarly, IgA1 were increased only in males, but not in female asthmatics, compared to controls. IgG2, but not IgG1, IgG3, and IgG4, was lower in asthmatics compared to healthy donors. This difference was significant in male but not in female asthmatics. In addition, IgG4 concentrations were reduced in females compared to male asthmatics. Considering the blocking activity of IgG4, the latter findings might explain why asthma tends to be more severe in women [9]. Total IgA and IgA1 were increased after menopause in female asthmatics. Serum concentrations of total IgA were increased in mild, but not severe asthmatics compared to controls, whereas IgE levels were increased in both groups of asthmatics. An inverse correlation between serum levels of IgA2 and IgE was found in asthmatics but not in controls. A positive correlation was found between IgG2 and both total IgA and IgA2 in asthmatics. Collectively, our results highlight a gender dimorphism in IgA subclasses in asthmatic patients.

Previous studies have examined the serum concentrations of total IgA in children [51–55] and adults with asthma [56–58]. Unfortunately, these studies have not evaluated the serum concentrations of IgA1 and IgA2 in both healthy controls and asthmatics. Moreover, these old studies did not examine the phenotype of asthmatic patients. In addition, serum concentrations of IgA increase with age [58, 59] and, therefore, results in children cannot be compared to those in adults. In a previous study on 15 normal subjects, 9 mild and 22 adults with severe asthma, serum IgA and IgG were decreased only in severe asthmatics compared to healthy controls [56]. These results apparently differ from our study in which we found that mild, but not severe T2-high asthmatics, had increased serum total IgA levels. In a population-based study, history of asthma was more prevalent in adults with selective IgA deficiency [57]. In a large population-based study of 1,136 adult patients with asthma, serum IgA increased with age and positively correlated to IgE [58]. To the best of our knowledge, our study is the first to evaluate the serum concentrations of total IgA, IgA1 and IgA2 in adult patients with T2-high asthma compared to age-matched healthy subjects. We found that serum levels of total IgA are increased in asthmatic subjects compared to controls, total IgA are lower in female asthmatics compared to males, and IgA1 subclass is increased in male asthmatics compared to controls. Steffen and collaborators have also demonstrated that serum IgA and IgA1, but not IgA2 are increased in healthy men compared to women [29].

Human IgG subclasses are similar in structure but differ in binding to receptors and accessory molecules, altering their functionality [60]. In this study, we found that serum levels of IgG2 were lower in asthmatics compared to controls. Moreover, IgG4 concentrations were decreased in females compared to male asthmatics. In a small number of asthmatics and healthy subjects, serum IgG in severe asthmatics were decreased when compared to controls [56]. By contrast, in a large study, allergic subjects had significantly higher IgG4 levels than controls and this difference was more pronounced for men than women [61].

IgG4 coexist as two isomers differing from other immunoglobulin subclasses in the disulfide bonding of hinge cysteines [34, 60]. In vivo, half-molecules of IgG4 recombine randomly with other half-molecules of IgG4, combining specificities of two molecules, resulting in monovalent-bispecific antibodies [62]. The resulting IgG antibody has low affinity for the activating Fc receptor for IgG (Fc*γ*R), while retaining high affinity for the inhibiting Fc*γ*RIIb. IgG, by interacting with Fc*γ*RIIb inhibits downstream signaling through Fc*ε*RI, thus preventing the release of proinflammatory mediators from basophils and mast cells [63]. IgG4 has therefore been characterized as “blocking antibody” in the context of allergic disorders [34]. The clinical relevance of lower concentrations of IgG4 in female compared to male asthmatics requires additional investigations.

Previous studies indicate that serum IgA levels increase with age [58, 59]. We found that serum concentrations of IgA are significantly correlated with the age of asthmatic patients. This observation prompted us to analyze total IgA, IgA1, and IgA2 in female asthmatics and controls before and after menopause. Interestingly, we found that total IgA and IgA1 are increased in postmenopausal asthmatic women. We cannot exclude the possibility that the alterations in total IgA and IgA1 levels found in post-menopausal asthmatic women are associated with the age of donors. However, the specificity of our observation is supported by the finding that serum IgA2 are not affected by menopause in female patients. This novel observation is not surprising because it is well-established that menopause has a distinct impact on the female immune system. For instance, several hormones (e.g., estradiol, testosterone, FH, FSH) can affect the immune responses [7, 64, 65]. At menopause, estradiol production in the ovaries ceases. Thereafter, only basal levels of progesterone are being synthesized by the adrenal glands. In aged women, dehydroepiandrosterone (DHEA) and testosterone levels decrease, yet follicle-stimulating hormone (FSH) and luteinizing hormone (LH) levels rise from the fourth decade onward [66]. In men, there is a slower yet steady decline in testosterone levels displaying no clear turning point [67]. For instance, female hormones can modulate IgA production in experimental models [68–70].

IgE is central to type I immediate allergic responses [31–33]. Allergen-specific IgA2 and polymeric IgA2 have been shown to be elevated following allergen immunotherapy [34, 71]. A positive correlation between serum IgA and IgE has been reported in adult asthmatics [58]. Our present study for the first time demonstrates an inverse correlation between serum IgE and IgA2 in asthmatics. Moreover, we found a positive correlation between IgG2 and both total IgA and IgA2 only in asthmatic patients. The latter observation is interesting because the types of antigens that could elicit IgG2 and IgA appear to be similar, as the majority of environmental antigens that induce IgA are glycosylated. Moreover, both IgA2 [27, 72] and IgG2 can form dimers [60]. Collectively, our results reveal a series of complex correlations between serum IgA2 and both IgE and IgG2 in asthmatics that require additional investigations.

Gender differences in serum IgA and IgA1 and to a lesser extent in IgG4 between male and female asthmatics could be explained by genetic, epigenetic and non-genetic factors (e.g., microbiota, lifestyle). At first sight, the findings that IgA, IgA1, and IgG4 are increased in male asthmatics *versus* females were surprising because nine genes corresponding to nine isotypes of heavy chains (i.e., *μ*, *δ*, *γ*1, *γ*2, *γ*3, *γ*4, *α*2 and *ε*) are located on chromosome 14 [73]. However, we cannot rule out the possibility that some of the ≅ 50 X-linked genes involved in the modulation of immunity [74] also dictate sex differences in immunoglobulin synthesis.

Epigenetic studies could help to address the molecular basis of disparities of serum IgA, IgA1, and IgG4 in male and female asthmatics. The possibility exists that sex hormones influence epigenetic factors (e.g., DNA methylation, chromatin conformation) [75, 76] that control immunoglobulin synthesis, thus playing a role in gender-dependent IgA, IgA1, and IgG4 disparities in male and female asthmatics.

Sex hormones, in particular androgens, seem critical in shaping the gut microbiota composition [77, 78]. There is growing evidence for gender dimorphism of gut microbiota in humans and other species [79–81], which may contribute to divergent development of the host immune system [82, 83]. Asthma development has been increasingly associated with gut and lung microbiome alterations [84, 85]. An aberrant IgA response to the gut microbiota precedes asthma development [86]. IgA regulates the composition and function of the gut microbiota and modulates its interaction with the host [20, 87]. Future studies should investigate the possible interactions between gut and lung microbiota, sex hormones and serum IgA subclasses in male and female asthmatics.

In adults, asthma is more prevalent and severe in women [9]. In addition, female patients experience more symptoms when compared to males [9]. Sex disparities in asthma remain poorly understood and could be influenced by lifestyle-associated allergy risk factors (e.g., exposure to cigarette smoke, diesel exhaust, detergents) [88]. It is difficult to control lifestyle in patient cohorts; however, the existence of lifestyle-dependent sex differences in IgA and IgG subclasses could be investigated by using appropriate experimental models of lung inflammation [27].

This study has several limitations that should be pointed out. The sample size of the asthma patient and healthy control cohorts investigated in the present study is limited. Further studies on larger cohorts of both healthy controls and asthmatics could better highlight the significance of gender dimorphism in IgA and IgG subclasses in these patients. For instance, we anticipate that it would be necessary to examine at least one order of magnitude larger cohorts to correlate alterations of serum IgA and IgG subclasses to lung function in asthmatic patients. Asthma is a heterogeneous group of inflammatory disorders characterized by distinct subtypes and variable clinical courses [35–38]. The patients included in this study were adults with a T2-high asthma phenotype of different severity (mild and severe). The limited sample size of patients did not allow to correlate the serum concentrations of IgA and IgG subclasses to T2-low or T2-high asthma [39, 89]. Finally, results might be confounded by glucocorticoid treatment [90], especially because the majority of the asthmatic participants were using high-dose ICS and some systemic glucocorticoids. The effects of high-dose ICS and systemic glucocorticoids on serum levels of IgA and IgG subclasses in asthmatics are not known.

## Conclusions

Our study showing different levels of gender dimorphism in IgA subclasses in asthmatic patients might have translational relevance in clinical manifestations of asthma. First, we found that IgA and IgA1 were markedly increased in males, but not female asthmatics compared to controls. Similarly, IgG4 were augmented in male compared to female asthmatics. Second, within the female asthma population, IgA and IgA1 were increased after menopause compared to premenopause. IgA antibodies play important roles in protecting subjects from bacterial and viral infections at mucosal surfaces, including in the airways [91–93]. In this study, we found that females have lower levels of IgA, IgA1, and IgG4 compared to male asthmatics. It is tempting to speculate that lower serum concentrations of these antibodies in female asthmatics could contribute to the increased prevalence and severity of asthma in adult women [9]. Interestingly, after menopause, asthma becomes more severe in males [10] and serum levels of IgA/IgA1 are higher in postmenopause compared to premenopause in female asthmatics.

The impact of gender on disease biology and treatment outcomes is well-appreciated in medical disciplines, such as cardiology [94, 95] and oncology [96–98], whereas its relevance in allergy was thus far underestimated in both clinical and preclinical studies. More adequate consideration of the immunological basis of gender disparity in asthma may open new opportunities in personalized medicine by optimizing diagnosis and targeted therapy.

## Supplementary Information

Below is the link to the electronic supplementary material.

Supplementary file1 (TIF 2233 kb)

Supplementary file2 (TIF 1988 kb)

Supplementary file3 (TIF 2007 kb)

Supplementary file4 (DOCX 384 kb)

## Data Availability

The datasets generated during and/or analyzed during the current study are available from the corresponding author on reasonable request.

## Abbreviations

ACT: Asthma control test
BMI: Body max index
ERS: European respiratory society
DHEA: Dehydroepiandrosterone
Fc*γ*R: Fc receptor for IgG
FSH: Follicle-stimulating hormone
FEV_1_: Forced expiratory volume in the 1st second
FEF_25–75_: Forced expiratory flow between 25 and 75% of FVC
FVC: Forced vital capacity
GCP: Good clinical practice
ICS: Inhaled glucocorticoids
Ig: Immunoglobulin
LABA: Long-acting *ß*-2 agonist
LAMA: Long-acting muscarinic agonist
LH: Luteinizing hormone
LTRA: Leukotriene-receptor antagonist
OCS: Oral glucocorticoids
pIgR: Poly-Ig receptor
PRED: Prednisone
SARP: Severe asthma research program
