# Supplementary figures and images for: Gender dimorphism in IgA subclasses in T2-high asthma
Source: Clin Exp Med. 2022 Apr 25;23(3):929–41. doi: 10.1007/s10238-022-00828-x (PMC10285012; doi:10.1007/s10238-022-00828-x)

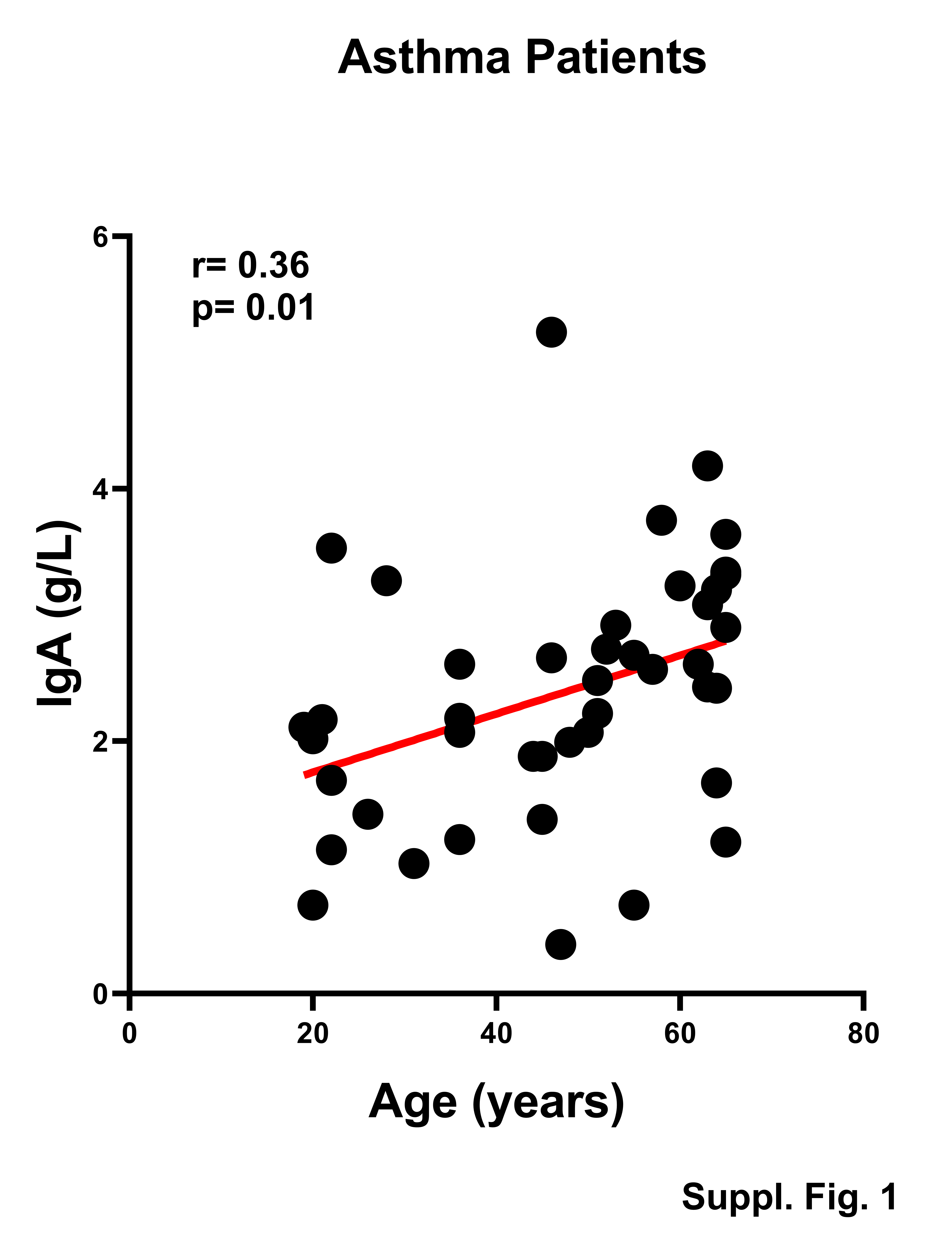

Supplement: Supplementary file 1 — Supplementary file1 (TIF 2233 kb) [file 10238_2022_828_MOESM1_ESM.tif]

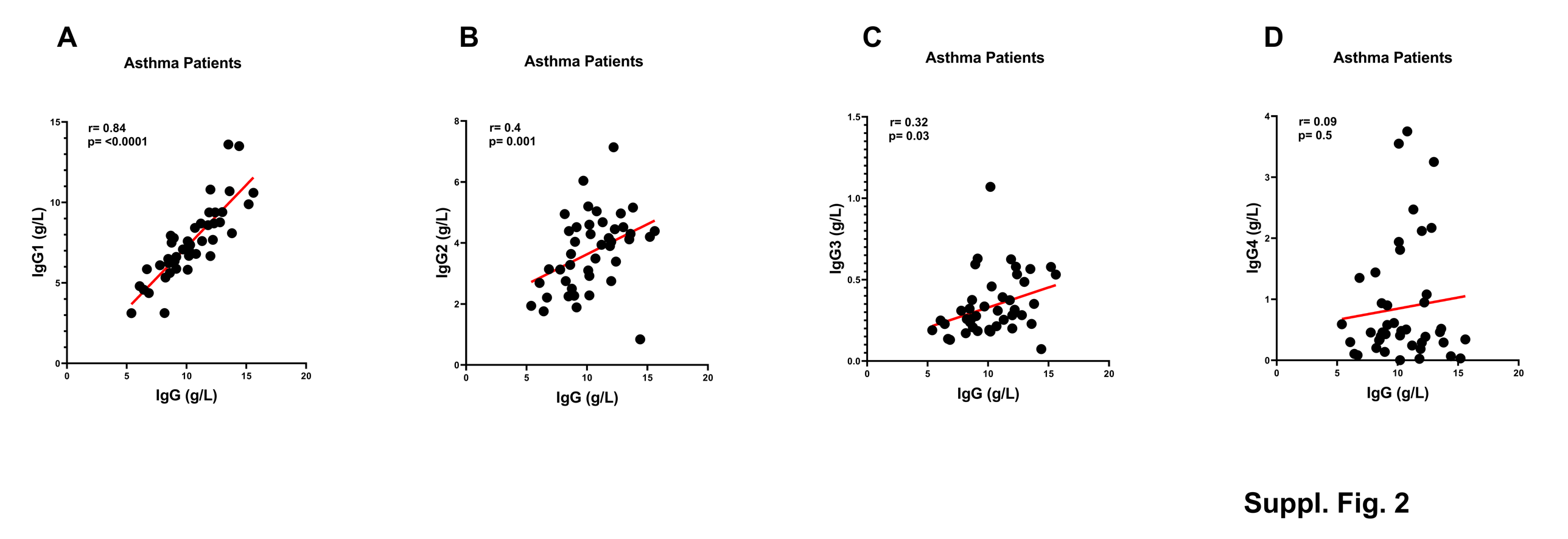

Supplement: Supplementary file 2 — Supplementary file2 (TIF 1988 kb) [file 10238_2022_828_MOESM2_ESM.tif]

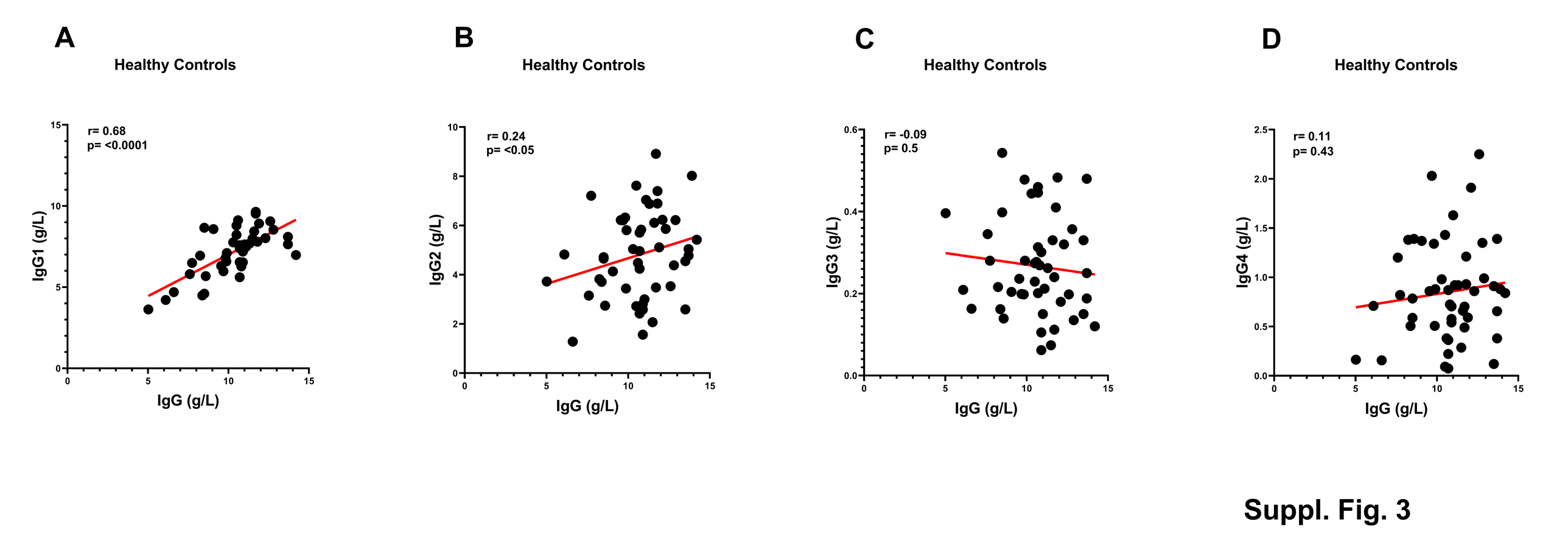

Supplement: Supplementary file 3 — Supplementary file3 (TIF 2007 kb) [file 10238_2022_828_MOESM3_ESM.tif]
