# Supplementary material for: Gender dimorphism in IgA subclasses in T2-high asthma
Source: Clin Exp Med. 2022 Apr 25;23(3):929–41. doi: 10.1007/s10238-022-00828-x (PMC10285012; doi:10.1007/s10238-022-00828-x)
Supplement: Supplementary file 4 — Supplementary file4 (DOCX 384 kb) [file 10238_2022_828_MOESM4_ESM.docx]

*Clinical and Experimental Medicine*

**ORIGINAL ARTICLE**

**Gender dimorphism in IgA subclasses in T2-high asthma**

Gilda Varricchi^1,2,3,4*☩^**·** Remo Poto^1,2,3☩^**·** Bianca Covelli^1^ **·** Gaetano Di Spigna^1^ **·** Maria Rosaria Galdiero^1,2,3,4^ **·** Gianni Marone^1,2,3,4^ **·** Loredana Postiglione^1,2,3^ **·** Giuseppe Spadaro^1,2,3*^

^1^ Department of Translational Medical Sciences, University of Naples Federico II, 80131 Naples, Italy

^2^ Center for Basic and Clinical Immunology Research (CISI), University of Naples Federico II, 80131 Naples, Italy

^3^ World Allergy Organization (WAO) Center of Excellence, 80131 Naples, Italy

^4^ Institute of Experimental Endocrinology and Oncology (IEOS), National Research Council, 80131 Naples Italy

***Corresponding authors**

Gilda Varricchi

[gildanet@gmail.com](mailto:gildanet@gmail.com)

Giuseppe Spadaro

[spadaro@unina.it](mailto:spadaro@unina.it)

**^☩^** Co-first authors (G.V.; R.P.)

**ORCID ID**

Gilda Varricchi 0000-0002-9285-4657

Remo Poto 0000-0002-4723-0167

Bianca Covelli 0000-0002-0980-4529

Gaetano Di Spigna 0000-0003-4590-1753

Maria Rosaria Galdiero 0000-0002-8086-9130

Gianni Marone 0000-0002-9849-4701

Loredana Postiglione 0000-0002-5382-8106

Giuseppe Spadaro 0000-0001-7889-425X

**SUPPLEMENTARY**

**Supplementary Fig. 1** Correlation between age of asthma patients and serum concentrations of IgA. Significance was tested with Pearson's correlation method.


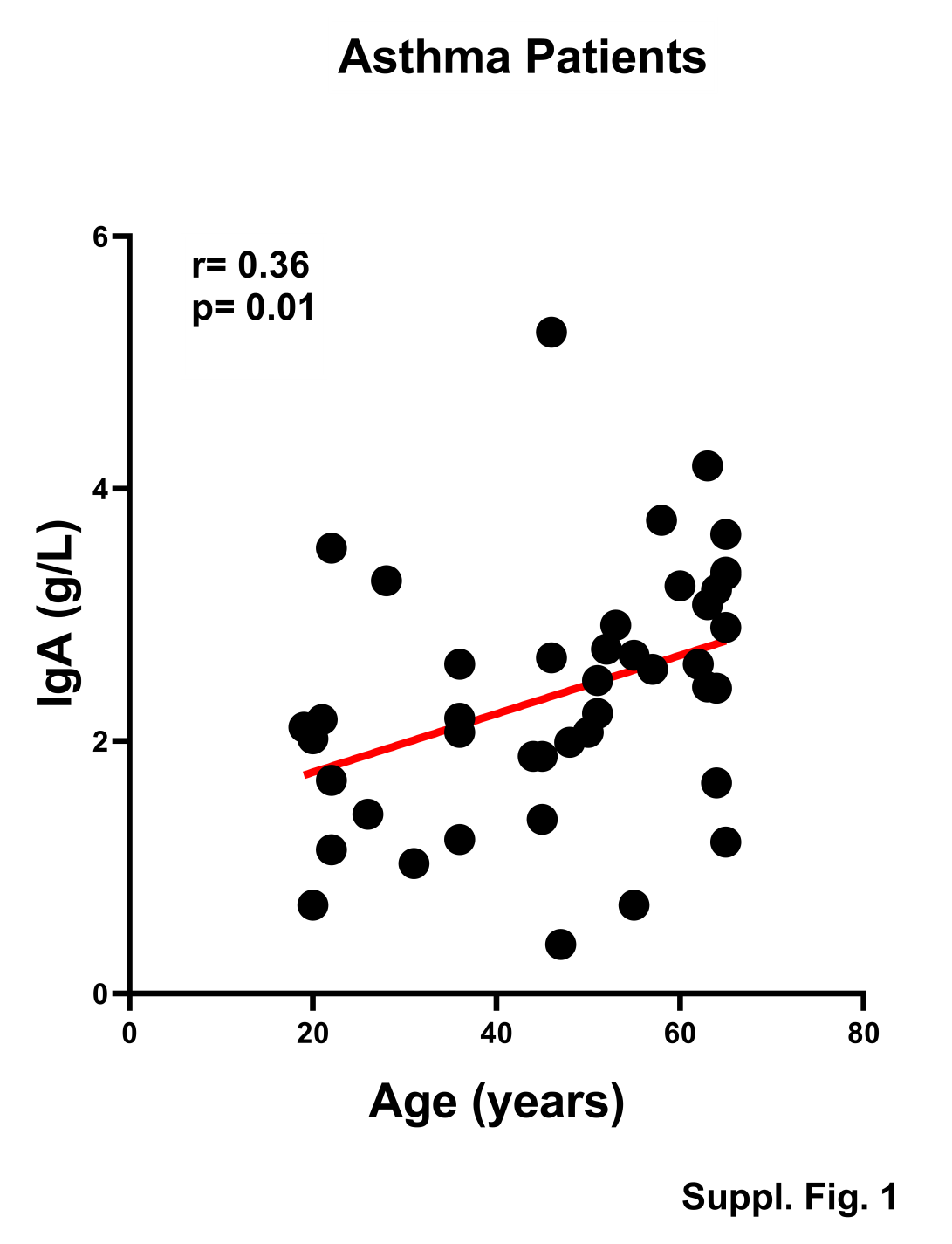


**Supplementary Fig. 2** Correlation between IgG and IgG1 (**A**), IgG2 (**B**), IgG3 (**C**), IgG4 (**D**), in asthma patients. Significance was tested with Pearson's correlation method.


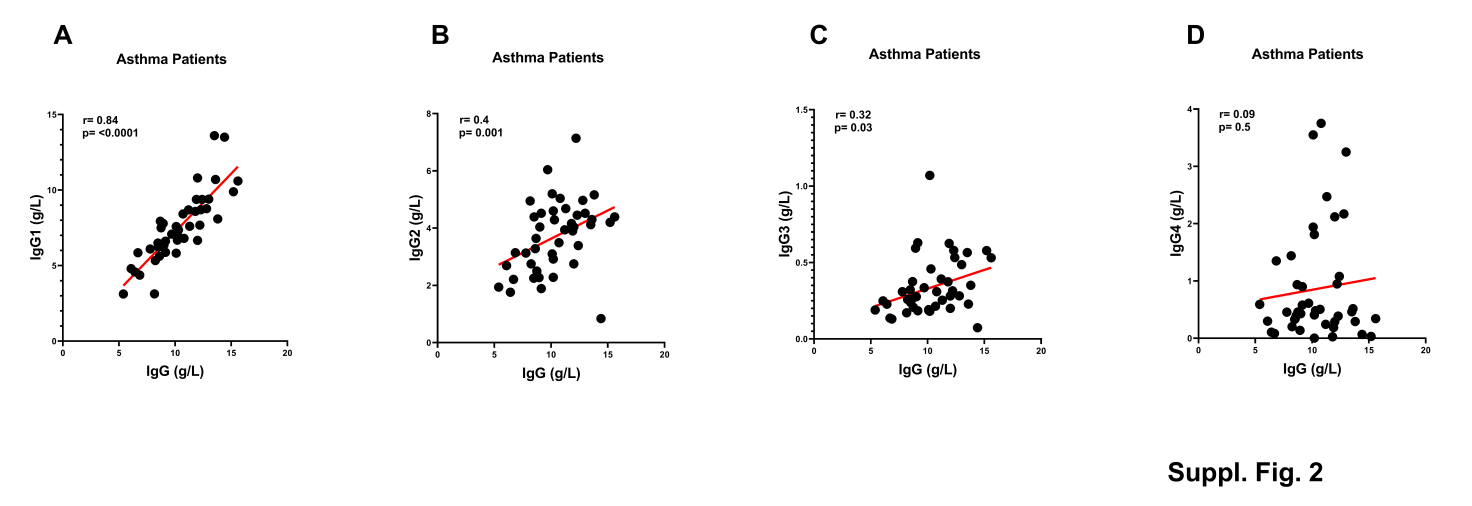


**Supplementary Fig. 3** Correlation between IgG and IgG1 (**A**), IgG2 (**B**), IgG3 (**C**), IgG4 (**D**), in healthy controls. Significance was tested with Pearson's correlation method.

**
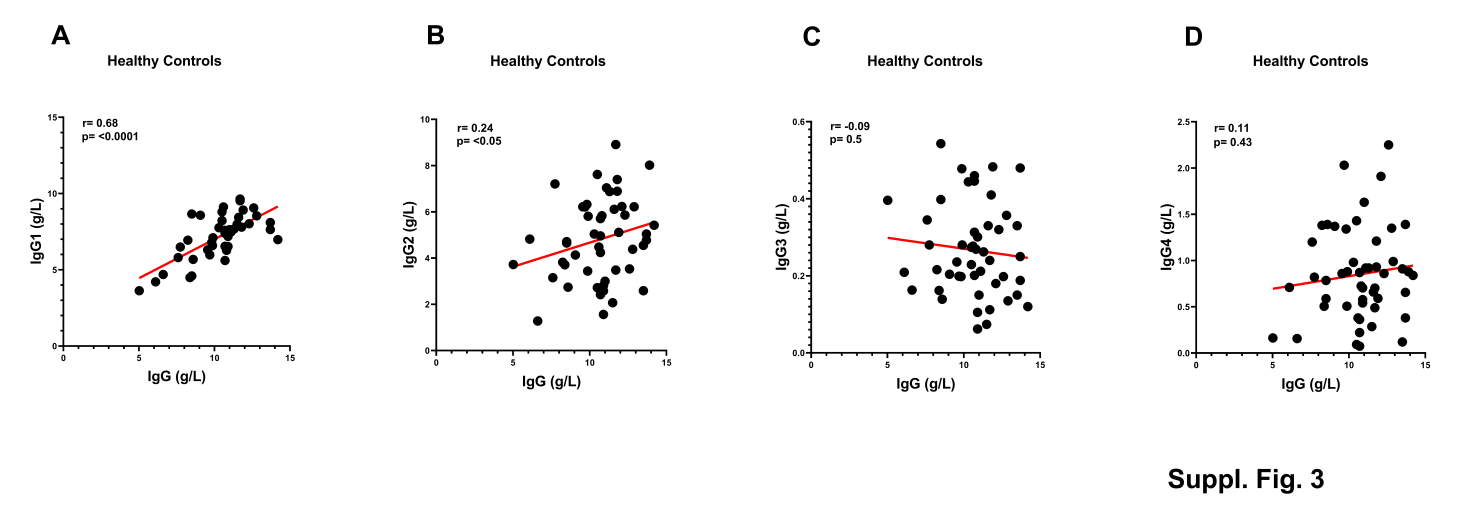
**
